# Prioritization of Candidate Biomarkers for Degenerative Aortic Stenosis through a Systems Biology-Based In-Silico Approach

**DOI:** 10.3390/jpm12040642

**Published:** 2022-04-15

**Authors:** Nerea Corbacho-Alonso, Tamara Sastre-Oliva, Cecilia Corros, Teresa Tejerina, Jorge Solis, Luis F. López-Almodovar, Luis R. Padial, Laura Mourino-Alvarez, Maria G. Barderas

**Affiliations:** 1 Department of Vascular Physiopathology, Hospital Nacional de Paraplejicos, SESCAM, 45071 Toledo, Spain; ncorbacho@sescam.jccm.es (N.C.-A.); tsastre@sescam.jccm.es (T.S.-O.); lmourino@sescam.jccm.es (L.M.-A.); 2 Department of Cardiology, Hospital Universitario 12 de Octubre, Instituto de Investigación Sanitaria Hospital 12 de Octubre (imas12), 28041 Madrid, Spain; ceciliacorros@yahoo.com (C.C.); jsolismartin@yahoo.es (J.S.); 3 Department of Pharmacology, School of Medicine, Universidad Complutense, 28040 Madrid, Spain; teje@med.ucm.es; 4 AtriaClinic, 28047 Madrid, Spain; 5 Centro de Investigación Biomédica en Red de Enfermedades Cardiovasculares (CIBERCV), Instituto de Salud Carlos III, 28029 Madrid, Spain; 6 Cardiac Surgery, Hospital Virgen de la Salud, SESCAM, 45004 Toledo, Spain; lopezalmodovar@yahoo.es; 7 Department of cardiology, Hospital Virgen de la Salud, SESCAM, 45004 Toledo, Spain; lrodriguez@sescam.org

**Keywords:** aortic valve, biomarkers, endoplasmic reticulum, in silico models, systems biology

## Abstract

Degenerative aortic stenosis is the most common valve disease in the elderly and is usually confirmed at an advanced stage when the only treatment is surgery. This work is focused on the study of previously defined biomarkers through systems biology and artificial neuronal networks to understand their potential role within aortic stenosis. The goal was generating a molecular panel of biomarkers to ensure an accurate diagnosis, risk stratification, and follow-up of aortic stenosis patients. We used in silico studies to combine and re-analyze the results of our previous studies and, with information from multiple databases, established a mathematical model. After this, we prioritized two proteins related to endoplasmic reticulum stress, thrombospondin-1 and endoplasmin, which have not been previously validated as markers for aortic stenosis, and analyzed them in a cell model and in plasma from human subjects. Large-scale bioinformatics tools allow us to extract the most significant results after using high throughput analytical techniques. Our results could help to prevent the development of aortic stenosis and open the possibility of a future strategy based on more specific therapies.

## 1. Introduction

Aortic stenosis (AS) is defined as an abnormal narrowing of the aortic valve (AV) opening, which blocks blood flow from the left ventricle into the aorta and, consequently, to the rest of the organism. The most common valve disease in the elderly is calcific or degenerative AS, which remains the main cause of AV replacement in developed countries [1,2,3].

AS progresses from an initial stage of aortic sclerosis, with a thickening and stiffening of the AV, to severe calcific stenosis. Unfortunately, the disease is usually diagnosed at an advanced stage since the symptoms are usually insidious at the onset. The appearance of its most common symptoms, such as dyspnea, angina, and syncope, predict a rapid deterioration of left ventricular function and the development of heart failure, potentially provoking the death of the patient if the pathology progresses. The only effective treatment to avoid this and improve survival is AV replacement, either surgically or via a transcatheter, which makes the management of these patients difficult [4,5]. As surgery should only be performed when the risks of AS outweigh those of the intervention, it is important to define different indicators to stratify the risk and timing of such interventions [6]. Early interventions may expose the patient to an unnecessary risk of complications, including living with a prosthetic valve and lifetime anticoagulation therapy, whereas an excessive delay may produce irreversible damage to the myocardium [7].

Ideally, the assessment of the global risk requires the integration of multiple biomarkers (including clinical factors) and an evaluation of molecular indicators belonging to independent pathways [8,9]. In an effort to identify suitable markers, large-scale analysis or -omics studies are powerful tools that enable panels of biomarkers to be defined that may later be assessed in patient cohorts. Combining and re-analyzing the results of multiple -omics studies through a systems biology approach allow AS treatment to be considered as a holistic process without applying a targeted hypothesis. As such, here, we used in silico studies that enabled us to combine results from our previous proteomics studies [10,11,12,13,14] with information from multiple databases, establishing a mathematical model thanks to the use of complex systems biology algorithms. Through this roadmap, we prioritized two proteins related to endoplasmic reticulum (ER) stress that have not been previously validated as markers for AS and analyzed them in a cell model as well as in plasma samples from human subjects.

## 2. Materials and Methods

### 2.1. Molecular Characterization of AS

**Figure 1:**
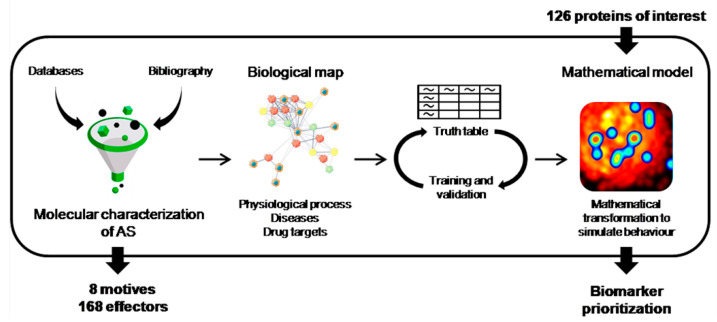
Mathematical model pipeline. Aortic stenosis was defined at molecular level through bibliography and database revision, a biological map was built, and mathematical models were trained. Then, candidate proteins were prioritized according to the functional relationship with the disease.

For the molecular characterization of AS, as well as the generation of mathematical models and candidate prioritization, an exhaustive bibliographic search of the molecular and cellular processes involved in the disease allowed the main pathophysiological events in AS (motives) to be identified and novel candidates to be defined (Figure 1). In this workflow, a search for reviews on the molecular pathogenesis and pathophysiology of the condition was performed in the PubMed database on 8 April 2019. The specific search was: (“degenerative aortic stenosis” [Title] OR “aortic stenosis” [Title] OR “calcific aortic valve disease”[Title] OR “calcific aortic stenosis” [Title]) AND (pathogenesis [Title/Abstract] OR pathophysiology [Title/Abstract] OR molecular [Title/Abstract]) and Review [ptyp]. Additionally, if the evidence of the implication of a candidate in the condition was judged not consistent enough to be assigned as an effector, an additional PubMed search was performed specifically for the candidate, including all the protein names according to UniProtKB.

### 2.2. Generation of the Mathematical Models 

To generate systems biology-based mathematical models, a biological map was built around the molecular processes and key proteins defined during the characterization of AS. The map was extended by adding knowledge-oriented connectivity layers (i.e., protein-to-protein interactions), including physical interactions and modulations, signaling and metabolic relationships, and the regulation of gene expression. Data were obtained from public and private databases (KEGG [15], BioGRID [16], IntAct [17], REACTOME [18], TRRUST [19], and HPRD [20]) and from manual curation of the relevant scientific literature. The models were then trained with a proprietary “Truth Table” containing publicly available data. The models must be able to weight the relative value of each protein (nodes), and since the number of links is very high, the number of parameters that must be resolved increases exponentially. The use of artificial intelligence technologies to model complex network behavior, including: graph theory and statistical pattern recognition technologies; genetic algorithms; artificial neural networks; dimensionality reduction techniques; and stochastic methods such assimulated annealing, Monte Carlo, etc.

### 2.3. Candidate Prioritization

The first step in candidate prioritization was the confection of a list of 126 proteins based on our previous studies (Table 1). Once the mathematical models had been generated, their predictive power can be exploited through an artificial neural network (ANN) strategy [21] in order to prioritize the different proteins and protein combinations based on their potential relationships with defined AS related processes (motives). Specifically, the potential relationship between each differentially expressed protein and the protein sets defining each AS motive (process) of interest was predicted through ANNs. This approach attempts to find the shortest distance between the protein sets, thereby generating a list of differentially expressed proteins ordered according to their association with the selected disease or pathway.

The ANNs evaluate the relationships among the protein sets or regions within the network, providing a predictive score that quantifies the probability a functional relationship exists between the network regions evaluated. Each score is associated with a *p*-value that describes the probability of the result being a true positive result. Three categories were used to group the proteins analyzed according to the predicted relationship value (Table 2): strongly related proteins (including the “Very high” group with a predicted ANN value ≥92% (*p < 0*.01), the “High” group with a predicted ANN value <92–≥78% (*p* values between 0.01 and 0.05), and the “Medium-high” group with a predicted ANN value <78–≥63% (*p* values between 0.05 and 0.15)); moderately related proteins (the “Medium” group with a predicted ANN value <63–≥38 (*p* values between 0.15 and 0.25)); and proteins with a low or no relationship (the “Low” group predicted ANN value <38% (*p* > 0.25)).

This classification defined those proteins predicted to have a:

- “strong relationship” with the processes under study, with very high, high or, medium-high predicted relationships with any of the sub-processes used in the characterization, and considered to be good candidates;
- “medium relationship” with the processes under study, with at least a medium predicted relationship with any of the sub-processes used in the characterization;
- “low or no relationship” with the processes under study and with a weak predicted relationship with all the sub-processes used in the characterization.

### 2.4. Cell Culture and Differentiation

Human cardiac valvular interstitial cells (HAVICs: Innoprot, P10462) were used in this study, cells isolated from heart valves, cryopreserved in primary cultures, and guaranteed to further expand for 10 population doublings under the conditions indicated in the data sheet. HAVICs were cultured in Fibroblast Medium-2 (FM-2: Innoprot), designed for optimal growth of normal human cardiac fibroblasts invitro, and containing essential and non-essential amino acids, vitamins, organic and inorganic compounds, hormones, growth factors, trace minerals, and a low concentration of fetal bovine serum (FBS, 5%). For the experiments, HAVICs were used at passage 5, and during the previous passage 4, the medium was replaced by a special medium for fibroblasts (FIBm) that favors a quiescent phenotype: Dulbecco’s Modified Eagle Medium (DMEM: Hyclone) supplemented with 2% heat-inactivated FBS, 150 U/mL penicillin-streptomycin, 2 mM L-glutamine, 10 ng/mL fibroblast growth factor (FGF-2), and 50 ng/mL insulin [22]. In the experiments, the cells were cultured for up 14 days in two different media, FIBm and osteogenic medium, to induce the osteogenic differentiation of the HAVICs (OSTm—FIBm supplemented with 50 µg/mL ascorbic acid, 10 mM β-glycerophosphate, and 100 nM dexamethasone) [23].

### 2.5. Alizarin Red Staining

The cells were washed with PBS, fixed with 4% paraformaldehyde for 15 min, and then incubated for 10 min with alizarin red S (Sigma Aldrich, St. Louis, MO, USA) [24]. After washing with deionized water, calcium deposition was visualized under an Olympus IX83 inverted microscope, capturing 49 images per well, and analyzing this with Scan^R software. These experiments were performed in triplicate.

### 2.6. Patient Selection and Plasma Extraction

Peripheral blood samples were collected from control subjects (*n* = 18) and from patients with severe AS (*n =* 18) who underwent follow-up at the Hospital 12 de Octubre (Madrid, Spain) and/or Hospital Virgen de la Salud (Toledo, Spain) from November 2018 to December 2019. All patients had severe AS diagnosed with two-dimensional echocardiography/doppler and were at least 50 years old. Control subjects were also subjected to echocardiographic control to avoid the presence of valve disease. Samples from patients with a severe morbidity (ischemic heart disease with ventricular dysfunction, end-stage chronic kidney disease), bicuspid AV, a family or personal history of aortopathy, rheumatic valve disease, and ≥moderate mitral valve disease were excluded from the study. Importantly, subjects were selected to avoid significant differences between the groups in terms of the main cardiovascular risk factors: gender, obesity, hypertension, dyslipidemia, and diabetes. Clinical characteristics of both groups are shown in Table 3.Blood samples (28 mL) were collected in tubes containing EDTA and centrifuged at 1125× *g* for 15 min, immediately freezing the resulting supernatant at −80 °C until analysis.

This study was carried out in accordance with the recommendations of the Helsinki Declaration, and it was approved by the Ethics Committee at the participant hospitals (approval reference numbers: 18/315 and 07/036). Signed informed consent was obtained from all subjects prior to their inclusion on the study.

### 2.7. Western Blotting

HAVICs were trypsinized and homogenized in lysis buffer containing protease inhibitors on day 7 or 14 of treatment [25]. The protein concentration of both the cell extracts and plasma samples was determined by the Bradford–Lowry method (Bio-Rad protein assay) [26]. Equal amounts of protein from the samples (10 µg for cell extracts and 25 µg for plasma) were resolved by SDS–PAGE in a Bio-Rad Miniprotean II electrophoresis cell run at a constant current of 25 mA/gel. After electrophoresis, the proteins were transferred to a nitrocellulose membrane under a constant voltage of 20V for 30 min, and the membranes were stained with Ponceau S to guarantee an equal amount of protein was loaded for each patient. Subsequently, the membranes were blocked for 1 h with PBS-Tween 20 (PBS-T) containing 7.5% non-fat dry milk and incubated overnight with the primary antibody in PBS-T with 5% non-fat dry milk. The primary antibodies used were antisera against thrombospondin-1 (THBS, 1/100, Abcam ab85762, Cambridge, UK), endoplasmin (GRP94, 1/100, Abcam ab3674, Cambridge, UK), and α-smooth muscle actin (SMA, 1/100, Abcam ab7817, Cambridge, UK). After washing, the membranes were incubated with a specific HRP-conjugated secondary antibody in PBS-T containing 5% non-fat dry milk, and antibody binding was detected by enhanced chemiluminescence (ECL: GE Healthcare), according to the manufacturers’ instructions. Densitometry was performed with the ImageQuantTL software (GE Healthcare). We used Ponceau S stain images to normalize Western blot data from cell cultures, a more consistent way of normalizing data than using a single house-keeping protein [27].

### 2.8. Statistics

Dichotomous variables are expressed as prevalence in number and percent, and continuous variables, such as age, are expressed as mean ± s.d. The normality of the data was assessed with the Kolmogorov–Smirnov test. Two-tailed Student t-tests were employed to calculate the differences between the groups and a general linear model adjusted for age was used to avoid the effect of age as confounder. All statistical analyses were performed using SPSS 15.0 for Windows software (SPSS Inc., Chicago, IL, USA). Statistical significance was accepted at *p* < 0.05.

## 3. Results

### 3.1. Molecular Motives of AS

After the bibliographic review of AS, eight pathophysiological processes or ‘motives’ were identified as being associated with this condition. These motives can be classified at two levels depending on their involvement in the pathology: causative, motives that are directly related to the onset or pathophysiology of the condition characterized; and symptomatic, motives that are a consequence of the pathology. Lipoprotein accumulation, inflammation, oxidative stress, endothelial dysfunction, oxidative stress, and the renin–angiotensin–aldosterone (RAA) system are all causatives motives in AS, whereashypertrophy and myocardial fibrosis are symptomatic. Calcification is included at both levels, as a cause and manifestation of the disease. The results of this search were thoroughly reviewed to identify protein/gene candidates that might be condition effectors, i.e., proteins whose activity (or lack thereof) is functionally associated witheach motive. A total of 168 proteins were defined as effectors of particular processes in AS or to AS in general (Table S2).

### 3.2. Candidate Prioritization

The mechanistic ANN ranking enabled the list of 126 proteins to be classified based on their predicted functional or mechanistic relationship. The ANN analysis indicated that, of the 126 candidate proteins, 61 (48.41%) were predicted to have a strong relationship with at least one process involved in degenerative AS or with degenerative AS in general (Table 4). Of these, 20 proteins are degenerative AS effectors already described in the molecular characterization of the disease, whereasthe remaining 41 proteins were not included in this characterization. Moreover, 32 of the 61 proteins are associated with more than one of the processes. The list of all proteins analyzed and the ANN score or relationship predicted values to the entire disease are presented in Supplementary Table S3. Whether the proteins are effectors of the disease is also displayed.

Moreover, there were 22 proteins strongly related to three or more of the processes evaluated, including general AS characterization (Table 5). Among these, eight proteins were not present in the molecular characterization: endoplasmin, decorin, alpha-2-macroglobulin, serum albumin, transthyretin, clusterin, and Thbs1.

### 3.3. Confirmation of the Prioritized Candidates in a Cell Model and Plasma

**Figure 2:**
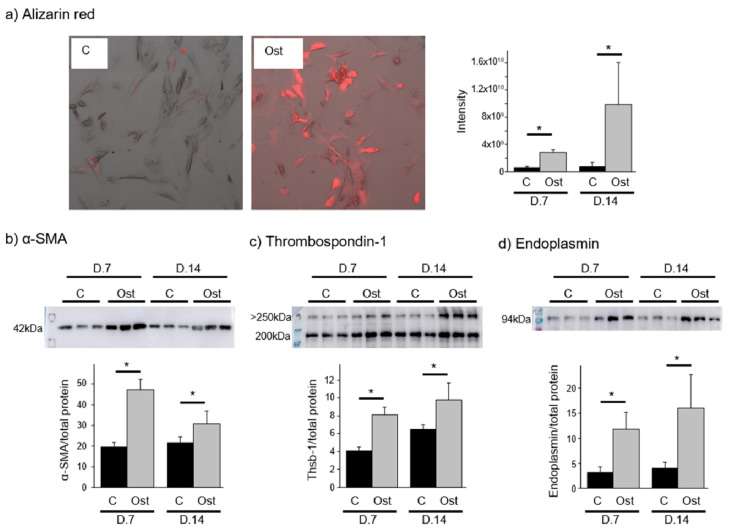
Verification of the osteoblastic differentiation through alizarin red staining (**a**) and α-SMA (**b**) and Western blot confirmation of thrombospondin-1 (**c**) and endoplasmin (**d**) levels in HAVICs treated with FIB medium (C) and osteogenic medium (Ost) after 7 and 14 days of culture. Data from western blots were normalized to total protein level (Ponceau S stain, Figure S1).*= *p <* 0.05.

Protein extracts from HAVICs were analyzed at 7 and 14 days of treatment, when higher levels of alizarin red and α-SMA were evident in the treated cells, confirming their osteoblastic differentiation (Figure 2a,b). There was also more total Thbs1 (day 7 *p*-value= 0.002; day 14 *p*-value = 0.045) and endoplasmin (day 7 *p*-value = 0.014; day 14 *p*-value = 0.038) in these HAVICs maintained in OSTm (Figure 2c,d). We found two different bands in the Western blot of Thbs1, one higher than 250 KDa (day 7, FIB medium = 0.681 ± 0.088, osteogenic medium = 1.268 ± 0.192, *p*-value = 0.009; and day 14, FIB medium = 0.862 ± 0.048, osteogenic medium = 2.527 ± 0.241, *p*-value = 0.005) and one at 200 KDa (day 7, FIB medium = 3.451 ± 0.458, osteogenic medium = 6.828 ± 0.968, *p*-value = 0.005; and day 14, FIB medium = 5.679 ± 0.467, osteogenic medium = 7.268 ± 1.708, *p*-value = 0.195), whichwere also analyzed separately.

**Figure 3:**
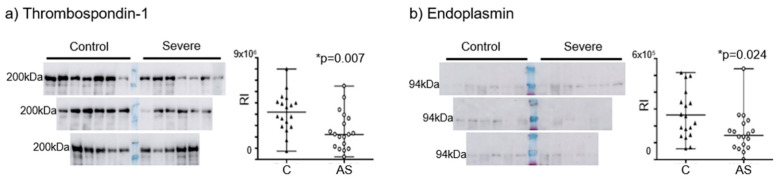
Western blot confirmation of thrombospondin-1 (**a**) and endoplasmin (**b**) levels in the plasma samples (control and severe AS subjects), with the corresponding *p*-values (Student’s *t*-test) for each protein analyzed: *= *p <* 0.05; RI, relative intensity.

The alterations to Thbs1 and endoplasmin were confirmed in Western blots of plasma from control subjects and severe AS patients. Consequently, we found lower levels of total Thbs1 (*p*-value = 0.007; age-adjusted *p*-value = 0.017) and endoplasmin (*p*-value = 0.024; age-adjusted *p*-value = 0.021) in the AS patients in both non-adjusted and age-adjusted model (Figure 3).

## 4. Discussion

Currently, there area large amount of data generated by high-throughput techniques such asproteomics, such that the interpretation and analysis of these data is becoming a complicated task. To overcome this challenge, systems biology approaches are essential, as they bring together all this information along with newly generated data. Systems biology uses a network-based approach to model complex biological systems and processes, employing mathematical models and computational approaches. These strategies allow new properties or mechanisms involved in a disease to be discovered that were not previously evident with traditional reductionist approaches [28].

In this work, systems biology approaches were used to evaluate and prioritize potential AS candidate biomarkers based on their association with the disease and their mechanistic implications. This ANN strategy provides a specific predictive value to the candidate markers identified, giving an idea of the probability that a relationship exists between each differentially expressed protein and the processes studied. This value is based on validating the predictive capacity of these models through the information available in the databases.

Our initial general characterization of AS identified six causative (calcification, lipoprotein accumulation, inflammation, oxidative stress, endothelial dysfunction, and RAA system) and two manifestation motives (hypertrophy and myocardial fibrosis). During AV degeneration, the causative motives are tightly related. In the initial phase, endothelial dysfunction occurs due to classic cardiovascular risk factors, such as advanced age, hypertension, smoking, diabetes mellitus, and the presence of high concentrations of cholesterol in the blood [29]. As a consequence, the permeability of the area increases, allowing the passage of molecules that leads to lipoprotein accumulation and inflammatory cytokine release. These lipids and cytokines further contribute to endothelial damage, amplifying the inflammatory process. In addition, this chronic inflammation causes oxidative stress, which, in turn, drives gene expression involved in the inflammatory process, thereby establishing a noxious vicious circle whereby inflammation causes oxidative stress and vice versa [30]. This activation of the immune system will provoke the differentiation of valvular interstitial cells from fibroblast to myofibroblasts, which will, in turn, develop angiogenic activity and produce a matrix of metalloproteins. The pro-inflammatory cytokines will induce the differentiation of a subgroup of myofibroblasts to osteoblasts, which leads to severe calcification and valve dysfunction [3,31]. Likewise, the RAA system plays an important role in the pathogenesis of AS. Its activation enhances collagen I and III mRNA expression, leading to myocardial fibrosis [32], and it is associated with left ventricle pressure overload. The combination of valve obstruction and elevated blood pressure imposes a high hemodynamic load on the left ventricle that leads to both left ventricle hypertrophy and myocardial fibrosis, two motives manifested in the general characterization of AS [33,34,35].

After the molecular characterization, and according to the mechanistic ANN ranking analysis, 22 proteins were found to be strongly related tothree or more of the processes evaluated. Of those 22 proteins, we highlight 8 of these that were not defined as effectors during the molecular characterization: decorin, alpha-2-macroglobulin, serum albumin, transthyretin, clusterin, endoplasmin and Thbs1. This study focused specifically on endoplasmin and Thbs1, as they are located in the ER. The ER is a major site for the regulation of calcium and lipid homeostasis, and it is essential for protein synthesis, folding, and transportation. When the influx of unfolded proteins to the ER exceeds its capacity to fold them correctly, unfolded and misfolded proteins accumulate in the ER lumen. This build-up creates a state defined as ER stress, and it activates a signaling pathway known as the unfolded protein response (UPR). In the context of AS, several studies indicate that oxidized low-density lipoprotein (oxLDL) causes ER stress in valvular interstitial cells by increasing cytosolic calcium levels [36,37]. Furthermore, oxLDL induces osteoblastic differentiation and promotes inflammatory responses via different ER stress-mediated pathways [38,39].

Endoplasmin, also known as glucose-regulated protein 94 (GRP94), HSP90b1, and gp96, is the most abundant glycoprotein in the ER and one of the major chaperones. Activation of the UPR results in the expression of genes encoding endoplasmin and other chaperones that mitigate the effects of increased load of unfolded proteins [40,41]. As all three branches of the UPR, the protein kinase-like ER kinase (PERK), inositol-requiring transmembrane kinase and endonuclease-1α (IRE1α), and activating transcription factor (ATF), are activated during bone formation to regulate expression of osteogenic genes, it is crucial to elucidate the role of endoplasmin in valve calcification [42,43,44,45,46]. Importantly, elevated levels of endoplasmin have been found in calcified vascular smooth muscle cells [47] and in the calcified aorta [48,49], consistent with our results.

Another protective mechanism in the calcified valve may be the increase in the levels of Thbs1, a multimeric Ca^2+^-binding glycoprotein that resides within the ER and that can be secreted by cells depending on the Ca^2+^ levels or the cell type examined [50]. As it matures in the ER, this protein also forms a complex with endoplasmin and other chaperones, such as PDI, BiP, and ERp72 [51], and it has the ability to mediate an ATF6α-dependent ER-stress response [52]. It has been suggested that Thbs1 is induced in the pressure-overloaded myocardium given that Thbs1−/− mice have greater cardiac hypertrophy than wild-type mice when submitted to pressure overload stimulation [53,54]. Our results are consistent with that phenomenon, and it seems that Thbs1 may act as a protective signal that prevents cardiac remodeling by altering fibroblast function and matrix metabolism. The appearance of two different protein isoforms of Thbs1 should also be further studied. It is known that this protein has a complex structure that includes a heparin-binding domain along with a procollagen homology domain at the amino terminus, and type I, II, and III repeats at the carboxyl-terminal end [55,56]. Thbs1 is implicated in several activities, such as homeostasis, apoptosis, or cell adhesion, as its domains can bind to receptors and specific proteins anchored in or secreted into the extracellular matrix [57,58,59,60]. As such, its synthesis and degradation are carefully regulated. Once secreted, the exposure of Thbs1 to specific microenvironmental milieus alters its structure and activity in a tissue and pathophysiological specific manner [61]. Several studies have found Thbs1 species of different molecular weights, and it has been suggested that this protein is rapidly cleared from circulation once secreted [62,63]. The influence of Thbs1 on cardiovascular diseases is complex and multifactorial, since its activity depends on the vessel type, the stage of the lesions, and associations with obesity, diabetes, or other metabolic diseases [64,65]. Thus, this protein should undoubtedly be further studied in the context of AS.

Confirmation of these proteins in both the cellular model and human plasma sample has different targets. Firstly, we used protein extracts from HAVICs submitted to osteogenic treatment. Although these proteins have previously been described in a small number of human samples, AS is a multifactorial disease and so it is difficult to discriminate if the alterations are due to the cardiocirculatory alterations caused by AV dysfunction or due to calcification itself. Moreover, AS patients are most often elderly and present different co-morbidities. These are the main limitations of this work: we have a small cohort and with different co-morbidities (although all related to cardiovascular disease). We have used a cohort of controls matched for risk factors, and we have excluded subjects with serious co-morbidities from the study, but we are aware that this may not be enough. All these drawbacks are partially avoided by the use of the cell model; this is not as complex a system as the organism, and thus the information obtained is not so complete. For this reason, in this work, we combined the insilico study and the cell model with an analysis of a larger cohort of patients to confirm the results. We searched for these proteins directly in plasma from healthy individuals and patients with severe AS. This step is important as it provides information about the usefulness of these proteins as diagnostic markers and may help translate the results to the clinical field, particularly as blood samples are easy to obtain and not too invasive compared to biopsies and surgical procedures. In the future, it would be interesting to quantify these proteins in a larger cohort, which will ideally allow the stratification of the subjects by age and co-morbidities. This will be an important step to improve precision medicine, as it will enable different thresholds to be established according to the specific characteristics of each patient, facilitating their management by clinicians.

## 5. Conclusions

In this work, we set out to demonstrate the importance of using largescale bioinformatics tools that allow us to consider all the data obtained through high-throughput analytical techniques to select the most significant results. Consequently, we will be able to select more specific targets and design future studies in a much more efficient way, better direct financial and social resources, and obtain higher quality results with a better chance of making advances and breakthroughs in our understanding and treatment of AS.

## Figures and Tables

**Table 1:** List of 126 proteins of interest based on our previous studies, showing the original work used for the selection of each protein. Additional information about their biological functions is shown in Table S1. These proteins were subsequently evaluated using the ANN strategy.

Protein Name	Uniprot ID	Reference	Protein Name	Uniprot ID	Reference
72 kDa type IV collagenase	P08253	Alvarez-Llamas G et al., 2013 [10]	Glutathione S-transferase P	P09211	Martin-Rojas T et al., 2012 [13]
Alcohol dehydrogenase 1B	P00325	Martin-Rojas T et al., 2015 [11]	Glycogen phosphorylase, liver form	P06737	Alvarez-Llamas G et al., 2013 [10]
Alpha-1-acid glycoprotein 1	P02763	Martin-Rojas T et al., 2015 [11]	Haptoglobin	P00738	Martin-Rojas T et al., 2012 [13]; Martin-Rojas T et al., 2015 [11]
Alpha-1-antichymotrypsin	P01011	Gil-Dones F et al., 2012 [12]; Alvarez-Llamas G et al., 2013 [10]	Hemoglobin subunit beta	P68871	Gil-Dones F et al., 2012 [12]
Alpha-1-antitrypsin	P01009	Martin-Rojas T et al., 2012 [13]; Martin-Rojas T et al., 2015 [11]; Gil-Dones F et al., 2012 [12]	Hemopexin	P02790	Martin-Rojas T et al., 2015 [11]; Gil-Dones F et al., 2012 [12]
Alpha-1B-glycoprotein	P04217	Martin-Rojas T et al., 2012 [13]	Histone H2A type 1-H	Q96KK5	Martin-Rojas T et al., 2015 [11]
Alpha-2-HS-glycoprotein	P02765	Martin-Rojas T et al., 2015 [11]; Gil-Dones F et al., 2012 [12]	Ig gamma-1 chain C region	P01857	Martin-Rojas T et al., 2015 [11]; Alvarez-Llamas G et al., 2013 [10]
Alpha-2-macroglobulin	P01023	Alvarez-Llamas G et al., 2013 [10]	Ig kappa chain C region	P01834	Gil-Dones F et al., 2012 [12]
Alpha-enolase	P06733	Martin-Rojas T et al., 2015 [11]	Ig lambda-1 chain C regions	P0CG04	Gil-Dones F et al., 2012 [12]
Angiotensinogen	P01019	Alvarez-Llamas G et al., 2013 [10]	Ig mu chain C region	P01871	Gil-Dones F et al., 2012 [12]
Annexin A1	P04083	Martin-Rojas T et al., 2015 [11]	Insulin-like growth factor-binding protein 5	P24593	Alvarez-Llamas G et al., 2013 [10]
Annexin A2	P07355	Martin-Rojas T et al., 2015 [11]	Insulin-like growth factor-binding protein 7	Q16270	Alvarez-Llamas G et al., 2013 [10]
Antithrombin-III	P01008	Gil-Dones F et al., 2012 [12]	Inter-alpha-trypsin inhibitor heavy chain H4	Q14624	Gil-Dones F et al., 2012 [12]
Apolipoprotein A-I	P02647	Martin-Rojas T et al., 2012 [13]; Gil-Dones F et al., 2012 [12]	Interleukin-6	P05231	Alvarez-Llamas G et al., 2013 [10]
Apolipoprotein A-IV	P06727	Gil-Dones F et al., 2012 [12]	Killer cell immunoglobulin-like receptor 3DL3	Q8N743	Alvarez-Llamas G et al., 2013 [10]
Apolipoprotein B-100	P04114	Alvarez-Llamas G et al., 2013 [10]	Kininogen-1	P01042	Gil-Dones F et al., 2012 [12]
Apolipoprotein C-II	P02655	Martin-Rojas T et al., 2015 [11]	Leucine-rich alpha-2-glycoprotein	P02750	Gil-Dones F et al., 2012 [12]
Apolipoprotein C-III	P02656	Gil-Dones F et al., 2012 [12]	Leukocyte receptor cluster member 9	Q96B70	Alvarez-Llamas G et al., 2013 [10]
Apolipoprotein E	P02649	Gil-Dones F et al., 2012 [12]	L-lactate dehydrogenase A chain	P00338	Martin-Rojas T et al., 2015 [11]
Beta-1,4-galactosyl- transferase 2	O60909	Alvarez-Llamas G et al., 2013 [10]	Lumican	P51884	Martin-Rojas T et al., 2012 [13]; Martin-Rojas T et al., 2015 [11]; Alvarez-Llamas G et al., 2013 [10]
Biglycan	P21810	Martin-Rojas T et al., 2015 [11]; Alvarez-Llamas G et al., 2013 [10]	Mannose-binding protein C	P11226	Gil-Dones F et al., 2012 [12]
Biogenesis of lysosome-related organelles complex 1 subunit 5	Q8TDH9	Martin-Rojas T et al., 2015 [11]	Metalloproteinase inhibitor 1	P01033	Alvarez-Llamas G et al., 2013 [10]
Calcineurin-binding protein cabin-1	Q9Y6J0	Martin-Rojas T et al., 2015 [11]	Metalloproteinase inhibitor 3	P35625	Alvarez-Llamas G et al., 2013 [10]
Calreticulin	P27797	Martin-Rojas T et al., 2012 [13]	Moesin	P26038	Martin-Rojas T et al., 2015 [11]
Cartilage oligomeric matrix protein	P49747	Alvarez-Llamas G et al., 2013 [10]	Nuclear factor NF-kappa-B p100 subunit	Q00653	Alvarez-Llamas G et al., 2013 [10]
Cathepsin B	P07858	Alvarez-Llamas G et al., 2013 [10]	Pentraxin-related protein PTX3	P26022	Alvarez-Llamas G et al., 2013 [10]
Cathepsin D	P07339	Alvarez-Llamas G et al., 2013 [10]	Peptidyl-prolyl cis-trans isomerase A	P62937	Martin-Rojas T et al., 2015 [11]
CD5 antigen-like	O43866	Gil-Dones F et al., 2012 [12]	Periostin	Q15063	Martin-Rojas T et al., 2015 [11]
CD9 antigen	P21926	Alvarez-Llamas G et al., 2013 [10]	Peroxiredoxin-1	Q06830	Martin-Rojas T et al., 2015 [11]
Ceruloplasmin	P00450	Gil-Dones F et al., 2012 [12]; Alvarez-Llamas G et al., 2013 [10]	Phosphoglycerate kinase 1	P00558	Martin-Rojas T et al., 2015 [11]
Chitinase-3-like protein 1	P36222	Alvarez-Llamas G et al., 2013 [10]	Phospholipid transfer protein	P55058	Alvarez-Llamas G et al., 2013 [10]
Chitinase-3-like protein 2	Q15782	Alvarez-Llamas G et al., 2013 [10]	Pigment epithelium-derived factor	P36955	Alvarez-Llamas G et al., 2013 [10]
Clusterin	P10909	Gil-Dones F et al., 2012 [12]; Alvarez-Llamas G et al., 2013 [10]	Plasma protease C1 inhibitor	P05155	Gil-Dones F et al., 2012 [12]; Alvarez-Llamas G et al., 2013 [10]
Coagulation factor XII	P00748	Gil-Dones F et al., 2012 [12]	Plasminogen activator inhibitor 1	P05121	Alvarez-Llamas G et al., 2013 [10]
Collagen alpha-1(III) chain	P02461	Alvarez-Llamas G et al., 2013 [10]	Pre-B-cell leukemia transcription factor-interacting protein 1	Q96AQ6	Alvarez-Llamas G et al., 2013 [10]
Collagen alpha-1(VI) chain	P12109	Martin-Rojas T et al., 2015 [11]; Alvarez-Llamas G et al., 2013 [10]	Procollagen C-endopeptidase enhancer 2	Q9UKZ9	Alvarez-Llamas G et al., 2013 [10]
Collagen alpha-1(XIV) chain	Q05707	Alvarez-Llamas G et al., 2013 [10]	Prolargin	P51888	Martin-Rojas T et al., 2015 [11]
Collagen alpha-2(I) chain	P08123	Alvarez-Llamas G et al., 2013 [10]	Prosaposin	P07602	Alvarez-Llamas G et al., 2013 [10]
Collagen alpha-3(VI) chain	P12111	Mourino-Alvarez L et al., 2016 [14]	Prostaglandin-H2 D-isomerase	P41222	Alvarez-Llamas G et al., 2013 [10]
Complement C1s subcomponent	P09871	Alvarez-Llamas G et al., 2013 [10]	Protein AMBP	P02760	Gil-Dones F et al., 2012 [12]
Complement C3	P01024	Gil-Dones F et al., 2012 [12]; Alvarez-Llamas G et al., 2013 [10]	Protein NDRG2	Q9UN36	Mourino-Alvarez L et al., 2016 [14]
Complement C4-A	P0C0L4	Gil-Dones F et al., 2012 [12]	Protein phosphatase 1 regulatory subunit 3E	Q9H7J1	Alvarez-Llamas G et al., 2013 [10]
Complement C4-B	P0C0L5	Gil-Dones F et al., 2012 [12]	Protein S100-A6	P06703	Martin-Rojas T et al., 2015 [11]
Complement component C9	P02748	Gil-Dones F et al., 2012 [12]	Prothrombin	P00734	Gil-Dones F et al., 2012 [12]
Complement factor H	P08603	Gil-Dones F et al., 2012 [12]	Serine protease HTRA1	Q92743	Alvarez-Llamas G et al., 2013 [10]
Complement factor H-related protein 1	Q03591	Gil-Dones F et al., 2012 [12]	Serotransferrin	P02787	Martin-Rojas T et al., 2015 [11]
Complement factor I	P05156	Gil-Dones F et al., 2012 [12]	Serum albumin	P02768	Martin-Rojas T et al., 2012 [13]; Martin-Rojas T et al., 2015 [11]
Cystatin-C	P01034	Alvarez-Llamas G et al., 2013 [10]	Serum amyloid P-component	P02743	Martin-Rojas T et al., 2012 [13]; Martin-Rojas T et al., 2015 [11]
Decorin	P07585	Martin-Rojas T et al., 2015 [11]	Serum paraoxonase/arylesterase 1	P27169	Gil-Dones F et al., 2012 [12]
EGF-containing fibulin-like extracellular matrix protein 1	Q12805	Alvarez-Llamas G et al., 2013 [10]	Serum paraoxonase/lactonase 3	Q15166	Alvarez-Llamas G et al., 2013 [10]
Endoplasmin	P14625	Martin-Rojas T et al., 2015 [11]	Spondin-1	Q9HCB6	Alvarez-Llamas G et al., 2013 [10]
Extracellular superoxide dismutase [Cu-Zn]	P08294	Martin-Rojas T et al., 2012 [13]	Superoxide dismutase [Cu-Zn]	P00441	Martin-Rojas T et al., 2015 [11]
Fatty acid-binding protein, adipocyte	P15090	Martin-Rojas T et al., 2012 [13]	Superoxide dismutase [Mn], mitochondrial	P04179	Martin-Rojas T et al., 2015 [11]
Fibrinogen alpha chain	P02671	Gil-Dones F et al., 2012 [12]	SWI/SNF complex subunit SMARCC1	Q92922	Alvarez-Llamas G et al., 2013 [10]
Fibrinogen beta chain	P02675	Gil-Dones F et al., 2012 [12]	Tenascin-X	P22105	Alvarez-Llamas G et al., 2013 [10]
Fibrinogen gamma chain	P02679	Martin-Rojas T et al., 2012 [13]; Gil-Dones F et al., 2012 [12]	Thrombospondin-1	P07996	Alvarez-Llamas G et al., 2013 [10]
Fibronectin	P02751	Alvarez-Llamas G et al., 2013 [10]	Transforming growth factor-beta-induced protein ig-h3	Q15582	Martin-Rojas T et al., 2015 [11]
Ficolin-2	Q15485	Gil-Dones F et al., 2012 [12]	Transgelin	Q01995	Martin-Rojas T et al., 2012 [13]; Martin-Rojas T et al., 2015 [11]
Follistatin-related protein 3	O95633	Alvarez-Llamas G et al., 2013 [10]	Transthyretin	P02766	Martin-Rojas T et al., 2012 [13]
FRAS1-related extracellular matrix protein 2	Q5SZK8	Alvarez-Llamas G et al., 2013 [10]	Triosephosphate isomerase	P60174	Martin-Rojas T et al., 2015 [11]
Galectin-1	P09382	Martin-Rojas T et al., 2015 [11]	Tubulin beta chain	P07437	Martin-Rojas T et al., 2015 [11]
Gelsolin	P06396	Alvarez-Llamas G et al., 2013 [10]	Vimentin	P08670	Martin-Rojas T et al., 2012 [13]; Martin-Rojas T et al., 2015 [11]
Glutathione peroxidase 3	P22352	Martin-Rojas T et al., 2012 [13]; Martin-Rojas T et al., 2015 [11]	Vitronectin	P04004	Gil-Dones F et al., 2012 [12]

**Table 2:** Category division of ANN score, in decreasing order, according to probability of being a true positive result.

ANN Category		ANN Score	Associated *p*-Value
**Strong relationship**	Very high	>92	<0.01
	High	78–92	0.01–0.05
	Medium-High	63–78	0.05–0.15
**Medium relationship**	Medium	38–63	0.15–0.25
**Low or no relationship**	Low	<38	>0.25

**Table 3:** Clinical characteristics of the subjects in the study: M/F, male/female; AHT, arterial hypertension; IHD, ischemic heart disease; BMI, body mass index.

	Controls	Severe AS	*p*-Value
**Age**	67.76 ± 10.03	79.94 ± 7.21	0.000
**Gender (M/F)**	11/7	9/9	0.584
**BMI**	28.48 ± 4.62	26.92 ± 3.67	0.282
**AHT (Yes,%)**	9 (50%)	12 (67%)	0.406
**Dyslipidemia (Yes, %)**	5 (28%)	9 (50%)	0.265
**Diabetes (Yes, %)**	0 (0%)	0 (0%)	1.000
**Smokers (Yes, %)**	2 (11%)	0 (0%)	0.584
**Pneumopathy (Yes, %)**	0 (0%)	1 (5%)	0.791
**Chronic IHD (Yes, %)**	0 (0%)	0 (0%)	1.000

**Table 4:** Categorization of the ANN score according to the probability of being a true positive result, showing the number of proteins with a strong relationship in each category. DAS, degenerative AS.

	Very High		High		Medium-High		
	Effectors	No Effectors	Effectors	No Effectors	Effectors	No Effectors	TOTAL
DAS general characterization	-	-	7	-	13	15	35
1. Calcification	-	-	4	1	1	4	10
2. Lipoprotein accumulation	-	-	-	-	2	10	12
3. Inflammation	-	-	3	5	1	11	20
4. Oxidative stress	-	-	0	1	3	3	7
5. Endothelial dysfunction	-	-	5	2	2	13	22
6. RAA system	-	-	-	-	1	4	5
7. Hypertrophy	-	-	1	1	-	8	10
8. Myocardial fibrosis	1	-	3	1	1	4	10

**Table 5:** Protein candidates that display a strong relationship with at least 3 degenerative AS motives, arranged in decreasing order of the highest ANN score. Proteins that were not described in the molecular characterization and are not considered effector proteins are in bold and highlighted.

Uniprot ID	Gene Name	Protein Name	Motive Effector	ANN Score	Related Motive
P08123	COL1A2	Collagen alpha-2(I) chain	Yes	92.52	Myocardial fibrosis
			No	90.86	Inflammation
			Yes	87.50	Endothelial dysfunction
			Yes	69.82	DAS General
P35625	TIMP3	Metalloproteinase inhibitor 3	Yes	91.85	Endothelial dysfunction
			Yes	82.53	DAS General
			No	69.20	Calcification
			No	63.61	RAA system
P02461	COL3A1	Collagen alpha-1(III) chain	Yes	91.69	Myocardial fibrosis
			Yes	87.29	Endothelial dysfunction
			Yes	76.47	DAS General
P01033	TIMP1	Metalloproteinase inhibitor 1	Yes	91.45	Endothelial dysfunction
			Yes	84.60	DAS General
			No	68.80	Calcification
P05231	IL6	Interleukin-6	Yes	90.45	Myocardial fibrosis
			Yes	88.05	Calcification
			Yes	87.64	Inflammation
			Yes	73.24	DAS General
P01042	KNG1	Kininogen-1	Yes	90.45	DAS General
			Yes	87.34	Inflammation
			No	70.10	RAA system
			No	63.40	Calcification
P07339	CTSD	Cathepsin D	Yes	87.00	DAS General
			Yes	84.46	Endothelial dysfunction
			No	71.66	Hypertrophy
P21810	BGN	Biglycan	Yes	86.97	Inflammation
			No	84.26	Calcification
			No	83.08	Myocardial fibrosis
			Yes	80.74	DAS General
			No	78.98	Endothelial dysfunction
			No	70.50	Lipoprotein accumulation
**P14625**	**HSP90B1**	**Endoplasmin**	**No**	**86.48**	**Inflammation**
			**No**	**74.62**	**Endothelial dysfunction**
			**No**	**70.58**	**DAS General**
			**No**	**65.44**	**Lipoprotein accumulation**
P01019	AGT	Angiotensinogen	Yes	84.81	DAS General
			Yes	82.54	Myocardial fibrosis
			Yes	78.60	Hypertrophy
			Yes	65.54	RAA system
P08253	MMP2	72 kDa type IV collagenase	Yes	84.40	Calcification
			Yes	75.40	Endothelial dysfunction
			Yes	74.10	Myocardial fibrosis
			Yes	73.98	DAS General
			No	71.88	Hypertrophy
			No	64.52	Oxidative stress
**P02766**	**TTR**	**Transthyretin**	**No**	**79.18**	**Oxidative stress**
			**No**	**74.11**	**Inflammation**
			**No**	**71.21**	**DAS General**
P00441	SOD1	Superoxide dismutase [Cu-Zn]	No	78.56	Inflammation
			Yes	74.41	DAS General
			Yes	69.12	Oxidative stress
**P10909**	**CLU**	**Clusterin**	**No**	**76.50**	**Inflammation**
			**No**	**67.68**	**Endothelial dysfunction**
			**No**	**64.01**	**Lipoprotein accumulation**
**P07996**	**THBS1**	**Thrombospondin-1**	**No**	**76.17**	**Endothelial dysfunction**
			**No**	**71.70**	**Myocardial fibrosis**
			**No**	**63.52**	**RAA system**
**P02768**	**ALB**	**Serum albumin**	**No**	**76.03**	**Inflammation**
			**No**	**75.65**	**Endothelial dysfunction**
			**No**	**68.55**	**Hypertrophy**
			**No**	**68.34**	**Lipoprotein accumulation**
Q00653	NFKB2	Nuclear factor NF-kappa-B p100 subunit	Yes	75.62	Inflammation
			Yes	74.83	Calcification
			Yes	72.15	DAS General
P04114	APOB	Apolipoprotein B-100	No	75.04	Endothelial dysfunction
			No	74.02	Inflammation
			Yes	71.61	DAS General
			Yes	64.60	Lipoprotein accumulation
P02647	APOA1	Apolipoprotein A-I	Yes	74.73	DAS General
			No	72.41	Endothelial dysfunction
			Yes	64.68	Lipoprotein accumulation
**P07585**	**DCN**	**Decorin**	**No**	**74.42**	**Calcification**
			**No**	**73.11**	**Endothelial dysfunction**
			**No**	**71.71**	**Hypertrophy**
			**No**	**68.53**	**Myocardial fibrosis**
**Q92743**	**HTRA1**	**Serine protease HTRA1**	**No**	**72.82**	**Myocardial fibrosis**
			**No**	**72.73**	**Endothelial dysfunction**
			**No**	**70.23**	**DAS General**
**P01023**	**A2M**	**Alpha-2-macroglobulin**	**No**	**72.55**	**Oxidative stress**
			**No**	**70.52**	**Myocardial fibrosis**
			**No**	**68.56**	**Inflammation**
			**No**	**68.30**	**Endothelial dysfunction**

## Data Availability

Not applicable.

## Supplementary Materials

The following supporting information can be downloaded at: https://www.mdpi.com/article/10.3390/jpm12040642/s1, Figure S1: Ponceau S stain image of the nitrocellulose membrane used for Western blots from cell cultures (a) and analysis of Thsb-1 (b) and endoplasmin (c) in plasma samples; Table S1: Functional analysis of the 126 proteins of interest selected from our previous studies. The proteins are represented in clusters according to their function. The enrichment score, number of terms and proteins included in each cluster are shown; Table S2: Effectors defined during the molecular characterization of the “Motives”: (1) calcification; (2) lipoprotein accumulation; (3) inflammation; (4) oxidative stress; (5) endothelial dysfunction; (6) RAA system; (7) hypertrophy; (8) myocardial fibrosis; Table S3: ANN score of each protein for each specific motive. The column effector indicates whether the protein was described in the molecular characterization, specifically in that motive [2,11,33,66,67,68,69,70,71,72,73,74,75,76,77,78,79,80,81,82,83,84,85,86,87,88,89,90,91,92,93,94,95,96,97,98,99,100,101,102,103].
