# Supplementary material for: Prioritization of Candidate Biomarkers for Degenerative Aortic Stenosis through a Systems Biology-Based In-Silico Approach
Source: J Pers Med. 2022 Apr 15;12(4):642. doi: 10.3390/jpm12040642 (PMC9026876; doi:10.3390/jpm12040642)

Table S2 Effectors defined during the molecular characterization of the "Motives": (1) calcification; (2) lipoprotein accumulation; (3) inflammation; (4) oxidative stress; (5) endothelial dysfunction; (6) RAA system; (7) hypertrophy; (8) myocardial fibrosis.

Gene name	Uniprot ID	Reference	Related motives	Gene name	Uniprot ID	Reference	Related motives
ACE2	Q9BYF1	Lindman BR et al, 2016 [18]; Sverdlov AL et al, 2011 [34]	6	MAP3K7	O43318	Nakamura M and Sadoshima J, 2018 [28]	7, 8
ACTA2	P62736	Perrucci GL et al, 2017 [33]	5	MAPK1	P28482	Pasipoularides A, 2016 [31]	7
ADIPOQ	Q15848	Kleinauskienė R and Jonkaitienė R, 2018 [14]	3	MAPK10	P53779	Nakamura M and Sadoshima J, 2018 [28]	7
ADRB1	P08588	Katholi RE and Couri DM, 2011 [13]	7	MAPK11	Q15759	Nakamura M and Sadoshima J, 2018 [28]	7
ADRB2	P07550	Adil SO et al, 2016 [1]; Nakamura M and Sadoshima J, 2018 [28]	7	MAPK12	P53778	Nakamura M and Sadoshima J, 2018 [28]	7
AGT	P01019	Lindman BR et al, 2016 [18]; Elmariah S and Mohler ER 3rd, 2010 [6]; Mathieu P et al, 2014 [24]; Nakamura M and Sadoshima J, 2018 [28]; Ma ZG et al, 2018 [20]	6, 7, 8	MAPK13	O15264	Nakamura M and Sadoshima J, 2018 [28]	7
AGTR1	P30556	Pasipoularides A, 2016 [31]; Ma ZG et al, 2018 [20]	6, 8	MAPK14	Q16539	Nakamura M and Sadoshima J, 2018 [28]	7
AGTR2	P50052	Lindman BR et al, 2016 [18]; Elmariah S and Mohler ER 3rd, 2010 [6]; Mathieu P et al, 2014 [24]; Katholi RE and Couri DM, 2011 [13]; Ma ZG et al, 2018 [20]	6, 7, 8	MAPK3	P27361	Pasipoularides A, 2016 [31]	7
AHSG	P02765	Kapelouzou A et al, 2015 [12]; Mathieu P and Boulanger MC, 2014 [23]	1	MAPK8	P45983	Nakamura M and Sadoshima J, 2018 [28]	7
AKAP6	Q13023	Nakamura M and Sadoshima J, 2018 [28]	7	MAPK9	P45984	Nakamura M and Sadoshima J, 2018 [28]	7
AKT1S1	Q96B36	Nakamura M and Sadoshima J, 2018 [28]	7	MATN2	O00339	García-Rodríguez C et al, 2018 [8]	1
ALPG	P10696	Lee SH and Choi JH, 2018 [16]	1	MEF2A	Q02078	Nakamura M and Sadoshima J, 2018 [28]	7
APOA1	P02647	O'Brien KD et al, 1996 [29]; Mathieu P et al, 2014 [24]	2	MGP	P08493	Doris MK et al, 2019 [5]; Mathieu P et al, 2014 [24]	1
APOB	P04114	O'Brien KD et al, 1996 [29]; Mathieu P et al, 2014 [24]	2	MLST8	Q9BVC4	Nakamura M and Sadoshima J, 2018 [28]	7
APOE	P02649	O'Brien KD et al, 1996 [29]	2	MME	P08473	Gallo G et al, 2019 [7]	3
BAK1	Q16611	Pasipoularides A, 2016 [31]	7	MMP14	P50281	Liu T et al, 2017 [19][19]	8
BAX	Q07812	Pasipoularides A, 2016 [31]	7	MMP2	P08253	Lee SH and Choi JH, 2018 [16]; Helske S et al, 2007 [9]; Liu T et al, 2017 [19]	1, 5
BCL2	P10415	Pasipoularides A, 2016 [31]	7	MMP9	P14780	Lee SH and Choi JH, 2018 [16]; Helske S et al, 2007 [9]	1, 5
BCL2L1	Q07817	Pasipoularides A, 2016 [31]	7	MSX2	P35548	Perrucci GL et al, 2017 [33]	1, 5
BGLAP	P02818	Cho KI et al, 2018 [3]	1	MTOR	P42345	Nakamura M and Sadoshima J, 2018 [28]	7
BGN	P21810	García-Rodríguez C et al, 2018 [8]	3	MYD88	Q99836	García-Rodríguez C et al, 2018 [8]; Zhan Q et al, 2017 [41]	1
BMP2	P12643	Kapelouzou A et al, 2015 [12]; Lindman BR et al, 2016 [18]; García-Rodríguez C et al, 2018 [8] ; Sverdlov AL et al, 2011 [34]	1	NFATC1	O95644	Lee SH and Choi JH, 2018 [16]; Nakamura M and Sadoshima J, 2018 [28]	1, 7

Gene name	Uniprot ID	Reference	Related motives	Gene name	Uniprot ID	Reference	Related motives
BMP4	P12644	Kapelouzou A et al, 2015 [12]; Lindman BR et al, 2016 [18]; García-Rodríguez C et al, 2018 [8]; Sverdlov AL et al, 2011 [34]	1	NFKB1	P19838	Sverdlov AL et al, 2011 [34] ; Lee SH and Choi JH, 2018 [16]	1, 3
BMP7	P18075	Osman L et al, 2006 [30]	1	NFKB2	Q00653	Sverdlov AL et al, 2011 [34] ; Lee SH and Choi JH, 2018 [16]	1, 3
CA12	O43570	Lindman BR et al, 2016 [18]; Myasoedova VA et al, 2018 [27]	1	NOS3	P29474	Towler DA, 2008 [36]; Mathieu P et al, 2014 [24]	4, 8
CAMK2D	Q13557	Nakamura M and Sadoshima J, 2018 [28]	7	NOTCH1	P46531	Lindman BR et al, 2016 [18]; Elmariah S and Mohler ER 3rd, 2010 [6]	1
CASP3	P42574	Mathieu P and Boulanger MC, 2014 [23]	1	NOX1	Q9Y5S8	Towler DA, 2008 [36]	4
CASP8	Q14790	Mathieu P and Boulanger MC, 2014 [23]	1	NOX4	Q9NPH5	Miller JD et al, 2008 [25]	4
CAT	P04040	Towler DA, 2008 [36]	4	NPPA	P01160	Gallo G et al, 2019 [7]	7. 8
CCL2	P13500	Lee SH and Choi JH, 2018 [16]	3	NPPB	P16860	Gallo G et al, 2019 [7]	7, 8
CD36	P16671	Syväranta S et al, 2014 [35]	3	NPPC	P23582	Gallo G et al, 2019 [7]	1, 8
CDH5	P33151	Perrucci GL et al, 2017 [33]	5	NR3C2	P08235	Katholi RE and Couri DM, 2011 [13]	7
CHP1	Q99653	Pasipoularides A, 2016 [31]	7	OLR1	P78380	Syväranta S et al, 2014 [35]	3
CMA1	P23946	Sverdlov AL et al, 2011 [34] ; Lindman BR et al, 2016 [18]; Helske S et al, 2007 [9]; Legere SA et 2019 [17]	6, 8	P2RY2	P41231	Lindman BR et al, 2016 [18]	1
COL1A1	P02452	Perrucci GL et al, 2017 [33]; Musa TA et al, 2018 [26]; Liu T et al, 2017 [19]	5, 8	PDE5A	O76074	Nakamura M and Sadoshima J, 2018 [28]	7
COL1A2	P08123	Perrucci GL et al, 2017 [33]; Musa TA et al, 2018 [26]; Liu T et al, 2017 [19]	5, 8	PDE9A	O76083	Nakamura M and Sadoshima J, 2018 [28]	7
COL3A1	P02461	Perrucci GL et al, 2017 [33]; Musa TA et al, 2018 [26]; Liu T et al, 2017 [19]	5, 8	PECAM1	P16284	Perrucci GL et al, 2017 [33]	5
CSF1	P09603	Lee SH and Choi JH, 2018 [16]	3	PLA2G7	Q13093	Capoulade R et al, 2014 [2]; Mathieu P and Boulanger MC, 2014 [23]	1, 2
CTGF	P29279	Ma ZG et al, 2018 [20]	8	PLCE1	Q9P212	Nakamura M and Sadoshima J, 2018 [28]	7
CTNNB1	P35222	Perrucci GL et al, 2017 [33]	5	POSTN	Q15063	Martin-Rojas T et al, 2015 [21]	1
CTSD	P07339	Helske S et al, 2007 [9]	5	POU1F1	P28069	Mathieu P et al, 2014 [24]; Mathieu P and Boulanger MC, 2014 [23]	1
CTSG	P08311	Sverdlov AL et al, 2011 [34]	6	PPARGC1A	Q9UBK2	Pasipoularides A, 2016 [31]	4
CTSK	P43235	Lee SH and Choi JH, 2018 [16]; Helske S et al, 2007 [9]	1, 5	PPP3CA	Q08209	Nakamura M and Sadoshima J, 2018 [28]	7
CTSL	P07711	Lee SH and Choi JH, 2018 [16]	1	PPP3R1	P63098	Nakamura M and Sadoshima J, 2018 [28]	7
CTSS	P25774	Lee SH and Choi JH, 2018 [16]; Helske S et al, 2007 [9]	1, 5	PRKACA	P17612	Mathieu P et al, 2014 [24]	1
CXCL8	P10145	Lee SH and Choi JH, 2018 [16]	3	PRKD1	Q15139	Nakamura M and Sadoshima J, 2018 [28]	7
CYBB	P04839	Towler DA, 2008 [36]; Miller JD et al, 2008 [25]	4	RELA	Q04206	Mathieu P et al, 2014 [24]; Lee SH and Choi JH, 2018 [16]	1, 3

Gene name	Uniprot ID	Reference	Related motives	Gene name	Uniprot ID	Reference	Related motives
CYC1	P08574	Pasipoularides A, 2016 [31]	7	RELB	Q01201	Sverdlov AL et al, 2011 [34] ; Lee SH and Choi JH, 2018 [16]	1, 3
DEPTOR	Q8TB45	Nakamura M and Sadoshima J, 2018 [28]	7	RETN	Q9HD89	Mathieu P and Boulanger MC, 2014 [23][23]	1, 4
DPP4	P27487	Cho KI et al, 2018 [3][3]	1	RPS6KB1	P23443	Nakamura M and Sadoshima J, 2018 [28]	7
EDN1	P05305	Peltonen T et al, 2008 [32]; Nakamura M and Sadoshima J, 2018 [28]; Liu T et al, 2017 [19]; Ma ZG et al, 2018 [20]	5, 7, 8	RPTOR	Q8N122	Nakamura M and Sadoshima J, 2018 [28]	7
EIF4E	P06730	Nakamura M and Sadoshima J, 2018 [28]	7	RUNX2	Q13950	Perrucci GL et al, 2017 [33]	1
EIF4EBP1	Q13541	Nakamura M and Sadoshima J, 2018 [28]	7	SELE	P16581	Cowell SJ et al, 2014 [4]; Lee SH and Choi JH, 2018 [16]	3, 5
ELN	P15502	Helske S et al, 2007 [9]	5	SIRT1	Q96EB6	Mathieu P and Boulanger MC, 2014 [23]	4
ENPP1	P22413	Mathieu P et al, 2014 [24]	1	SMAD2	Q15796	Nakamura M and Sadoshima J, 2018 [28]; Ma ZG et al, 2018 [20]	8
ENPP2	Q13822	Lindman BR et al, 2016 [18]	1	SMAD3	P84022	Nakamura M and Sadoshima J, 2018 [28]; Ma ZG et al, 2018 [20]	8
EPAC1	O95398	Nakamura M and Sadoshima J, 2018 [28]	7	SOD1	P00441	Towler DA, 2008 [36]	4
FGF1	P05230	Katholi RE and Couri DM, 2011 [13]; Liu T et al, 2017 [19]	7, 8	SOD2	P04179	Towler DA, 2008 [36]	4
GATA4	P43694	Nakamura M and Sadoshima J, 2018 [28]	7	SOD3	P08294	Towler DA, 2008 [36]	4
GRK2	P25098	Nakamura M and Sadoshima J, 2018 [28]	7	SP7	Q8TDD2	Lee SH and Choi JH, 2018 [16]	1
GRK5	P34947	Nakamura M and Sadoshima J, 2018 [28]	7	SPP1	P10451	Lee SH and Choi JH, 2018 [16];Cho KI et al, 2018 [3]	1
HDAC1	Q13547	Nakamura M and Sadoshima J, 2018 [28]	7	TGFB1	P01137	Osman L et al, 2006 [30]; Perrucci GL et al, 2017 [33]; Katholi RE and Couri DM, 2011 [13]; Nakamura M and Sadoshima J, 2018 [28]; Ma ZG et al, 2018 [20]	1, 5, 7, 8
HDAC2	Q92769	Nakamura M and Sadoshima J, 2018 [28]	7	TGFB3	P10600	Osman L et al, 2006 [30]	1
HDAC3	O15379	Nakamura M and Sadoshima J, 2018 [28]	7	TGFBR1	P36897	Nakamura M and Sadoshima J, 2018 [28]; Ma ZG et al, 2018 [20]	8
HDAC4	P56524	Nakamura M and Sadoshima J, 2018 [28]	7	TGFBR2	P37173	Nakamura M and Sadoshima J, 2018 [28]; Ma ZG et al, 2018 [20]	8
HDAC5	Q9UQL6	Nakamura M and Sadoshima J, 2018 [28]	7	TIMP1	P01033	Helske S et al, 2007 [9]	5
HDAC9	Q9UKV0	Nakamura M and Sadoshima J, 2018 [28]	7	TIMP2	P16035	Helske S et al, 2007 [9]	5
HMGB1	P09429	Weber C and Noels H, 2011 [38]	3	TIMP3	P35625	Helske S et al, 2007 [9]	5
IBSP	P21815	Kaden JJ et al, 2004 [11]	1	TIMP4	Q99727	Helske S et al, 2007 [9]	5
ICAM1	P05362	Venardos N et al, 2014 [37]; Lee SH and Choi JH, 2018 [16]	3, 5	TLR2	O60603	Lindman BR et al, 2016 [18]; Elmariah S and Mohler ER 3rd, 2010 [6]; García-Rodríguez C et al, 2018 [8]	1, 3

Gene name	Uniprot ID	Reference	Related motives	Gene name	Uniprot ID	Reference	Related motives
IGF1	P05019	Katholi RE and Couri DM, 2011 [13]	7	TLR3	O15455	Lee SH and Choi JH, 2018 [16]	1, 3
IL10	P22301	Kolasa-Trela R et al, 2017 [15]	3	TLR4	O00206	Lindman BR et al, 2016 [18]; Elmariah S and Mohler ER 3rd, 2010 [6]; García-Rodríguez C et al, 2018 [8]	1, 3
IL1A	P01583	Liu T et al, 2017 [19]	8	TNC	P24821	Kapelouzou A et al, 2015 [12]; Yetkin E, Waltenberger J, 2009 [39]	1
IL1B	P01584	Lee SH and Choi JH, 2018 [16]; Liu T et al, 2017 [19]	3, 8	TNF	P01375	Mathieu P et al, 2014 [24]; Lee SH and Choi JH, 2018 [16]; Mathieu P et al, 2014 [24]; Lin CP et al, 2015; Liu T et al, 2017 [19]	1, 2, 3, 4, 8
IL6	P05231	Mathieu P et al, 2015 [22]; Lee SH and Choi JH, 2018 [16]; Liu T et al, 2017 [19]	1, 3, 8	TNFRSF11A	Q9Y6Q6	Yip CY and Simmons CA, 2011 [40]; Izquierdo-Gómez MM et al, 2017 [10]	1
INS	P01308	Katholi RE and Couri DM, 2011 [13]	7	TNFRSF11B	O00300	Yip CY and Simmons CA, 2011 [40]; Izquierdo-Gómez MM et al, 2017 [10]	1
KNG1	P01042	Gallo G et al, 2019 [7]	3	TNFSF11	O14788	Yip CY and Simmons CA, 2011 [40]; Izquierdo-Gómez MM et al, 2017 [10]	1
LEP	P41159	Kolasa-Trela R et al, 2011	4, 5, 7	VCAM1	P19320	Cowell SJ et al, 2014; Lee SH and Choi JH, 2018 [16]	3, 5
LOX	P28300	Liu T et al, 2017 [19]	8	VIM	P08670	Perrucci GL et al, 2017 [33]	5
LPA	P08519	Lindman BR et al, 2016 [18]; Mathieu P et al, 2014 [24]	2	VWF	P04275	Perrucci GL et al, 2017 [33]	5
MAP2K3	P46734	Nakamura M and Sadoshima J, 2018 [28]	7	WNT11	O96014	Lee SH and Choi JH, 2018 [16]; Cho KI et al, 2018 [3]	1
MAP2K4	P45985	Nakamura M and Sadoshima J, 2018 [28]	7	WNT3A	P56704	Lee SH and Choi JH, 2018 [16]	1
MAP2K6	P52564	Nakamura M and Sadoshima J, 2018 [28]	7	WNT5A	P41221	Lee SH and Choi JH, 2018 [16]; Cho KI et al, 2018 [3]	1
MAP2K7	O14733	Nakamura M and Sadoshima J, 2018 [28]	7	WNT5B	Q9H1J7	Lee SH and Choi JH, 2018 [16]; Cho KI et al, 2018 [3]	1

Figure S1. Ponceau S stain image of the nitrocellulose membrane used for Western blots from cell cultures (**a**) and analysis of Thsb-1 (**b**) and endoplasmin (**c**) in plasma samples.

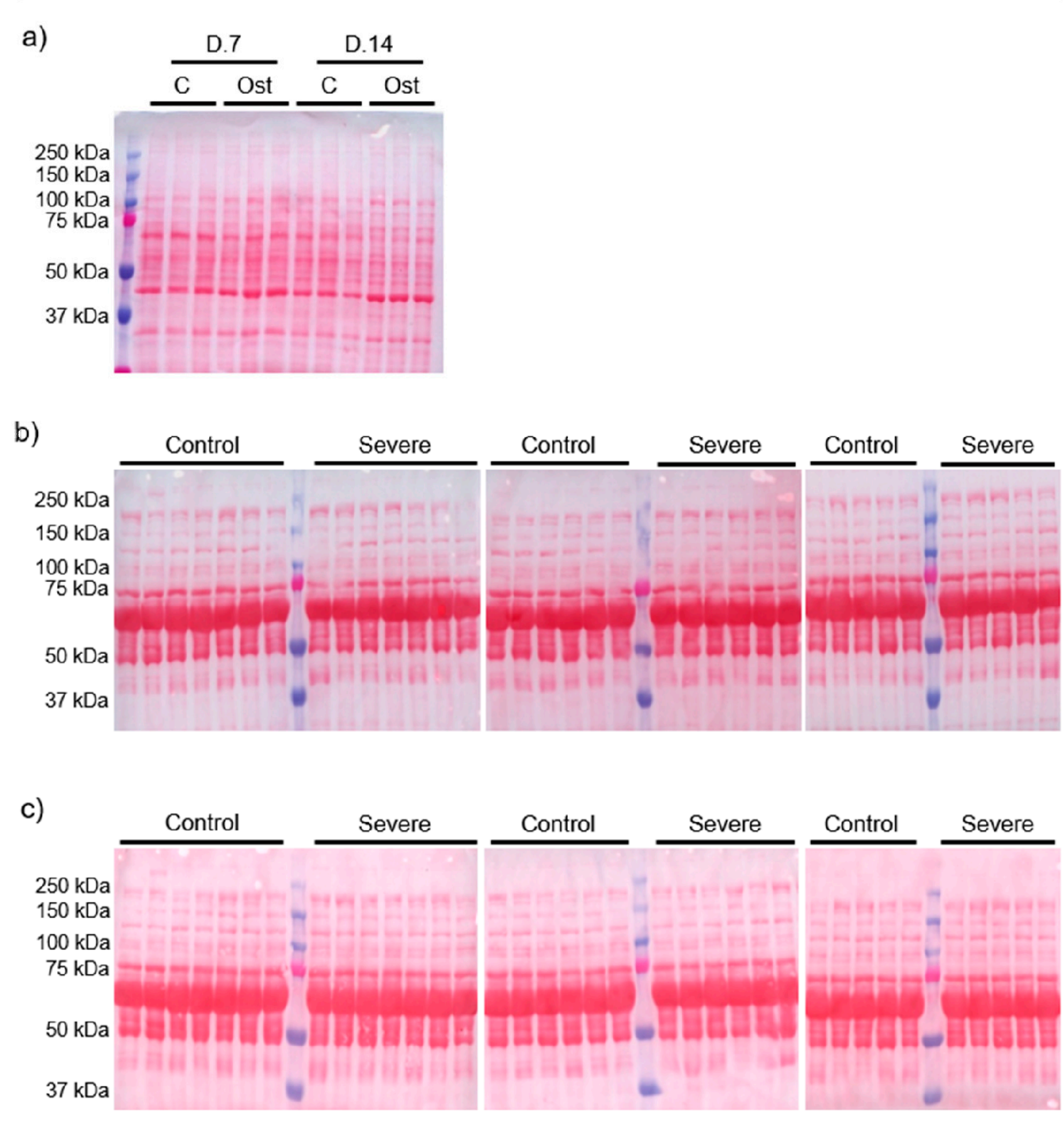

Supplement: Supplementary file 1 [file jpm-12-00642-s001.zip › jpm-1645448-supplementary/supplementary/Supplementary material_Rev.pdf]
